# Dermatoscopy-guided therapy of pigmented basal cell carcinoma with imiquimod*

**DOI:** 10.1590/abd1806-4841.20165255

**Published:** 2016

**Authors:** Husein Husein-ElAhmed, Maria Antonia Fernandez-Pugnaire

**Affiliations:** 1 Hospital de Guadix – Granada, Spain; 2 San Cecilio University Hospital – Granada, Spain

**Keywords:** Basal cell nevus syndrome, Drug therapy, Skin neoplasms

## Abstract

**BACKGROUND:**

Dermatoscopy is a non-invasive diagnostic tool used to examine skin lesions with an optical magnification. It has been suggested as a useful tool for monitoring therapeutic response in lentigo maligna patients treated with imiquimod.

**OBJECTIVE:**

To examine the accuracy of dermatoscopy as a tool to monitor the therapeutic response of pigmented basal cell carcinoma treated with imiquimod.

**METHOD:**

The authors designed a prospective study. Patients with pigmented basal cell carcinoma were included and data regarding the dermatoscopy features were collected following the Menzies criteria, prior to initiating the imiquimod treatment. Subsequent dermatoscopic evaluations were performed at weeks 4 and 8, following imiquimod discontinuation.

**RESULTS:**

Twenty lesions were included. The most common pigmented dermatoscopy features were large blue-grey ovoid nests (80%), followed by blue-grey globules (50%) and leaf-like areas (30%). No spoke wheel areas were observed. In 17 out of 20 patients, a response was noted during the first evaluation at 4 weeks, while the clearance was noted at the second check-up after 8 weeks. In two patients, the clearance was found at the initial evaluation at 4 weeks, while in one patient, the response remained unchanged. Blue-grey globules were the fastest to exhibit clearance (50% at week 4), followed by leaf-like areas (15%) and large blue-grey ovoid nests (6.25%).

**CONCLUSION:**

According to our results, dermatoscopic evaluation enhances the accuracy in the assessment of the clinical response to imiquimod in pigmented basal cell carcinoma.

## INTRODUCTION

Basal cell carcinoma (BCC) is the most prevalent malignant skin cancer originating from the basal cell layer of the epidermis. It rarely metastasizes and a proportion of these tumors may contain pigment. Most of the histological subtypes of basal cell carcinoma can exhibit pigmented varieties but this is rarely observed in morphoeic and infiltrative subtypes.^1^ Histologically, melanin can be found in the tumour cells and surrounding stroma. Within the tumour mass, melanin is more often seen in the superficial component of the tumour, with melanosomes often confined to the melanocytes. However, they may be taken up by the surrounding malignant epithelial cells.^2-4^ In the stroma, melanin is typically found at the tumour shoulders, in melanophages, but small amounts may lie free. Occasionally, melanocytes are found in the deep tumour nodules or in the overlying epidermis.^2^

Because of its asymmetry of pigmentation and variety of growth patterns, pigmented basal cell carcinoma (PBCC) is included in the differential diagnosis of invasive melanoma, along with other benign, pigmented skin lesions.

Dermatoscopy is a non-invasive diagnostic tool used to examine skin lesions with an optical magnification. Using a polarized light, this technique allows a detailed examination of pigmented epidermis structures and the dermo-epidermal junction. Its use has become highly important among dermatologists in conducting a better clinical diagnosis of nearly all pigmented lesions.^5-15^

Regarding pigmented basal cell carcinoma (PBCC), dermatoscopy has proven to be an effective diagnostic technique^2^. Most basal cell carcinomas have < 50% of their area pigmented, and only 7% of lesions have > 75% of pigmented tumour area.^16^

Although dermatoscopy has already been used as a tool to control borders in the surgical management of BCC, to the authors' knowledge, it has not yet been used to control accurately the efficacy of topical treatments such as imiquimod.^17^ This drug is an imidazoquinoline amide, which modifies immune response with both antitumor and antiviral activity. Its use was first approved in 2004 by the US Food and Drug Administration to treat actinic keratosis and superficial basal cell carcinoma.^18^ In addition, nodular subtypes of basal cell carcinoma have been treated with this cream, entailing positive responses.^19-23^ In the current literature, only one study reports a case of large PBCC, which was successfully resolved after imiquimod therapy.^24^

This study aims to examine the accuracy of dermoscopy as a tool for monitoring the therapeutic response of PBCC treated with imiquimod.

## METHOD

### Study design

The authors designed a study, conducted between January 1^st^, 2011 and May 1^st^, 2011. It was a prospective, open-label trial. The Menzies criteria were followed to diagnose PBCC; Absence of pigment network and at least, one of the following features: leaf-like areas, spoke wheel areas, large blue-gray ovoid nests and multiple blue-gray globules).^12^ BCCs manifesting arborizing vessels and/or ulceration, with no pigmentation features, were excluded. The authors included adult patients with dermatoscopy-confirmed diagnosis of PBCC.

### Imiquimod therapy

The treatment protocol for the lesions included in our study was the dosing frequency recommended by the manufacturer: one daily application 5 days a week for 6 weeks. Rest periods were used as necessary to manage local skin reactions, and continued for up to 6 weeks.

### Assessment of dermatoscopic features

Prior to initiating treatment, the team scanned the lesion using the Fotofinder^®^ device and collected data regarding the dermatoscopy features, following the above-mentioned criteria. Subsequent evaluations were performed at weeks 4 and 8 after imiquimod discontinuation. The team compared the images before and after to assess the changes in the dermatoscopy features. Responses were classified as: clearance (complete clearance of features), response (decrease in number and/or intensity of features), unresponsive (no changes in features) and worsening (increase in number and/or intensity of features).

The study's protocol was approved by the hospital's medical ethics committee, and all participants gave their written informed consent before enrollment.

## RESULTS

Twenty lesions from 20 subjects (9 women and 11 men) were included in the study. The median age was 68.73 (±9.11) years. Ten lesions were superficial BCC, and ten were of the nodular type. The most commonly affected site was the temple (5 lesions, 25%) followed by the nose (4 lesions, 20%). Table 1 summarizes the epidemiological data from our sample.

**Table 1:** Distribution of the sample with data regarding sex, BCC clinical type and location

Patient	Age	Sex	Clinical Type (Superficial / Nodular)	Location
1	76	M	S	Nose
2	81	M	S	Chest
3	72	F	N	Nose
4	74	M	N	Temple
5	68	F	S	Arm
6	81	M	N	Cheek
7	74	M	S	Temple
8	81	F	S	Forehead
9	93	M	S	Neck
10	74	F	N	Temple
11	69	M	S	Arm
12	71	M	N	Nose
13	73	F	S	Temple
14	62	F	N	Cheek
15	58	F	N	Trunk
16	70	F	N	Forehead
17	71	M	S	Ear
18	67	F	S	Temple
19	55	M	N	Nose
20	67	M	N	Trunk

All patients completed the 6-week treatment cycle. Application site reactions were the most common adverse effects, seen in 5 patients (25%). No patient required discontinuation of treatment or a rest period for local site reactions. In 17 out of 20 patients, a response was observed at the first evaluation after 4 weeks, while the clearance was noticed at the second check-up after 8 weeks. In two patients, the clearance was found at the first evaluation after 4 weeks (Patients 7 and 8), while in one patient (Patient 6), the response remained unchanged at the evaluation after 8 weeks, in relation to the evaluation after 4 weeks. No worsening was observed in our sample.

The most common pigmented dermatoscopic features were large blue-grey ovoid nests (16 lesions, 80%), followed by blue-grey globules (10 lesions, 50%) and leaf-like areas (6 lesions, 30%). Arborizing vessels were found in 13 lesions (65%) and ulcerations in 8 lesions (40%). No spoke wheel areas were observed.

Regarding large blue-grey ovoid nests, complete dermatoscopic clearance was observed at week 8 in most lesions (14 out of 16, 87.5%). Only in one lesion (6.25%) was the clearance observed at week 4, while in another lesion (6.25%), the response remained unchanged.

In 5 out of 10 lesions (50%), the blue-grey globules exhibited complete dermatoscopic clearance at week 8, while in the other 5 lesions (50%), the clearance was observed at week 4.

Leaf-like areas cleared completely by week 8 in 5 out of 6 lesions (85%), while in other lesions (15%), clearance was noted at week 4.

As regards arborizing vessels, complete dermatoscopic clearance was observed at week 8 in most lesions (9 out of 13, 69%). In 3 lesions (23%), the clearance was observed at week 4. In one lesion (8%), the response remained unchanged.

With respect to ulcerations, complete dermatoscopic clearance was noted at week 4 in all lesions (100%).

**Figure 1:**
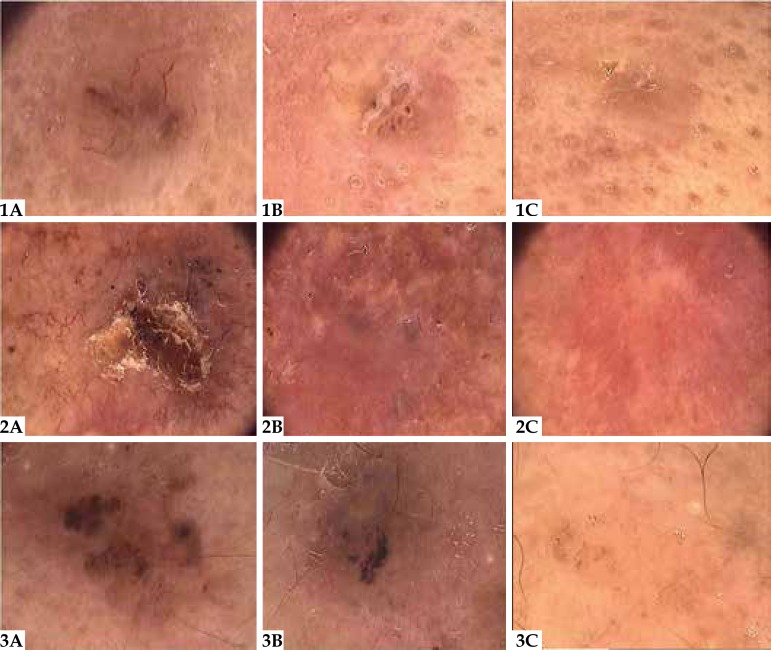
Dermatoscopic findings in three lesions: (A) at baseline; (B) 4 weeks after treatment discontinuation, showing a decrease in number and/ or intensity of features; (C) 8 weeks after treatment discontinuation, showing complete clearance of features. (Original magnification x 20)

Figure 1 displays changes in dermatoscopic features in three of the lesions at the baseline, at 4 weeks and 8 weeks after treatment discontinuation. No PBCC relapses were observed in the follow-up period (12 months in most cases). Table 2 outlines the dermatoscopic features observed in the lesions and the response to imiquimod cream at weeks 4 and 8, respectively.

**Table 2:** Dermatoscopic features of the 20 lesions prior to initiation of therapy and at weeks 4 and 8 after treatment with imiquimod

	ARBORIZING VESSELS	ULCERATION	BLUE-GRAY GLOBULES	OVOID NESTS	LEAF-LIKE AREAS	SPOKE WHEEL AREAS
Patient 1	+	-	+	**+**	**-**	-
W#4	R		R	**R**		
W#8	C		C	**C**		
Patient 2	-	-	-	**-**	**+**	-
W#4					**R**	
W#8					**C**	
Patient 3	+	-	+	**+**	**-**	-
W#4	R		C	**R**		
W#8	C		C	**C**		
Patient 4	+	+	+	**+**	**-**	-
W#4	C	C	C	**R**		
W#8	C	C	C	**C**		
Patient 5	-	+	+	**+**	**+**	-
W#4		C	C	**R**	**C**	
W#8		C	C	**C**	**C**	
Patient 6	+	-	-	**+**	**-**	-
W#4	R			**R**		
W#8	R			**R**		
Patient 7	+	-	-	**+**	**-**	-
W#4	C			**C**		
W#8	C			**C**		
Patient 8	+	-	+	**-**	**-**	-
W#4	C		C			
W#8	C		C			
Patient 9	-	+	+	**+**	**-**	-
W#4		C	R	**R**		
W#8		C	C	**C**		
Patient 10	-	-	+	**-**	**+**	
W#4			R		**R**	
W#8			C		**C**	
Patient 11	-	+	+	**-**	**+**	-
W#4		C	R		**R**	
W#8		C	C		**C**	
Patient 12	+	-	+	**+**	**-**	-
W#4	C		C	**R**		
W#8	C		C	**C**		
Patient 13	-	-	-	**+**	**+**	-
W#4				**R**	**R**	
W#8				**C**	**C**	
Patient 14	+	-	-	**+**	**+**	-
W#4	C			**R**	**R**	
W#8	C			**C**	**C**	
Patient 15	+	-	-	**+**	**-**	-
W#4	C			**R**		
W#8	C			**C**		
Patient 16	+	+	+	**+**	**-**	-
W#4	C	C	R	**R**		
W#8	C	C	C	**C**		
Patient 17	-	+	-	**+**	**-**	-
W#4		C		**R**		
W#8		C		**C**		
Patient 18	+	-	-	**+**	**-**	-
W#4	R			**R**		
W#8	C			**C**		
Patient 19	+	+	-	**+**	**-**	-
W#4	C	C		**R**		
W#8	C	C		**C**		
Patient 20	+	+	-	**+**	**-**	-
W#4	C	C		**R**		
W#8	C	C		**C**		

R: Response of features (decrease in number and/or intensity). C: clearance (complete clearance of features)

## DISCUSSION

Dermatoscopy is a non-invasive technique of *in vivo* microscopy, used to diagnose non-pigmented and pigmented skin lesions by allowing the visualization of morphologic structures that are usually not discernible to the naked eye. In addition to this diagnostic role, it seems to be helpful in defining the margin of pigmented lesions such as lentigo maligna and it has been suggested as a useful tool for monitoring therapeutic response in PBCC patients treated with imiquimod.^25-28^ In this study, the authors evaluated the efficacy of dermatoscopy in monitoring the treatment of PBCC with imiquimod, based on the lesions' dermatological features. The changes in specific dermatoscopic features of BCC after treatment are of interest. Among these features, those with pigmentation according to the Menzies method are: large blue-gray ovoid nests, blue-grey globules, maple leaf-like areas and spoke wheel areas. The authors noted a rapid clearance of all pigmentation signs and signs of neovascularization and ulceration.

Ulceration and neovascularization were the first dermatoscopic features to undergo complete clearance in our study. Regarding pigmented dermatoscopic features, blue-grey globules were the fastest to exhibit clearance (50% at week 4), followed by leaf-like areas (15%) and large blue-grey ovoid nests (6.25%). Onan *et al.* have correlated the dermatoscopic findings of PBCC with their histological features.^29^ According to these authors, blue-grey globules correlate with small tumour nests localized in the papillary dermis, leaf-like areas correlate with multifocal tumour nests connecting each other localized in the papillary dermis, and blue-grey ovoid nests correlate with well-bordered tumour nests, with a few small buddings at the periphery, localized on the papillary and/or reticular dermis. Based on the findings of the study, the authors suggest that the onset and order of clearance of the pigmented dermatoscopic features are linked to the depth and size of the histological structures, with more intense and quicker imiquimod effects on the smaller structures localized at superficial layers. However, this is a purely morphological observation.

Clinical response does not always match the clearance of PBCC. The dermatoscopic assessment of clearance in the study suggests that dermatoscopy may have a place in monitoring the topical treatment of these lesions. At week 4, most dermatoscopic features had not undergone complete clearance, which may be interpreted as persistent PBCC when evaluated clinically. It was with the second dermatoscopic evaluation at week 8, when additional signs decreased and complete clearance of dermatoscopic features were apparent and the clearance was noticed. This outcome is of great interest to avoid clinical misdiagnosis with persistent PBCC. Dermatoscopic assessment provides more accuracy and precision to match the clearance of PBCC treated with imiquimod.

Topical, non-invasive, patient-administered treatment modalities continue to expand the options of dermatologists in managing a variety of skin conditions, including skin cancers. Less patient discomfort, favorable cosmetic outcomes and documented efficacy against BCCs make imiquimod an attractive treatment choice for managing PBCC. Imiquimod therapy plays a role in PBCC patients where other invasive treatment modalities are not recommended. Poor surgical candidates (i.e., patients who are elderly, anticoagulated or who have implanted cardiac pacemakers) would benefit from this non-invasive, self-administered topical therapy. In this context, dermatoscopy is a helpful tool for evaluating the clearance of the lesion after imiquimod without the need for assessing histological remission with risky incisional biopsies. Nonetheless, since dissociation between clinical and histological clearance has been reported, biopsies are still required to confirm remission. Indeed, the main limitation of our study is that no biopsies were performed to assess histological remission.^30,31^ However, a biopsy does not allow examination of the whole lesion because specimens are usually obtained from representative areas of a lesion to predict the histopathological condition. Hence, the authors suggest that dermatoscopic evaluation of treated lesions may enhance the accuracy of treatment response assessments: if dermatoscopic features remain on the treated lesion, a biopsy should be performed on that site. In this study, no signs of lesion recurrence were observed after one-year of follow-up and therefore no biopsies were needed.

Randomized controlled studies comparing dermatoscopic findings, as well as a histopathological evaluation of complete surgical excision, are required to confirm the real usefulness of dermatoscopy in monitoring imiquimod therapy for PBCC.

To the authors' knowledge, this is the first study to examine dermatoscopy as a therapeutic tool for monitoring PBCC treated with imiquimod. In conclusion, the data suggest that dermatoscopy is not only a diagnosis technique, but that it may also be useful in managing PBCC by facilitating the monitoring of response to topical treatments.

## CONCLUSION

According to the findings, dermatoscopic evaluation enhances the accuracy in the assessment of the clinical response to imiquimod in PBCC.
